# Assessment of a Day Hospital Management Program for Children With Type 1 Diabetes

**DOI:** 10.1001/jamanetworkopen.2020.0347

**Published:** 2020-03-03

**Authors:** Sarah Lawson, Jacob M. Redel, Allison Smego, Melanie Gulla, Pamela J. Schoettker, Mary Jolly, Farida Mostajabi, Lindsey Hornung

**Affiliations:** 1 Cincinnati Children's Hospital Medical Center, Division of Pediatric Endocrinology, University of Cincinnati College of Medicine, Cincinnati, Ohio; 2 The University of Utah, Salt Lake City; 3 James M. Anderson Center for Health Systems Excellence, Cincinnati Children's Hospital Medical Center, University of Cincinnati College of Medicine, Cincinnati, Ohio; 4 Cincinnati Children's Hospital Medical Center, Division of Biostatistics & Epidemiology, University of Cincinnati College of Medicine, Cincinnati, Ohio

## Abstract

**Importance:**

A shift in the setting of care delivery for children with a new diagnosis of type 1 diabetes led to a reorganization of treatment.

**Objective:**

To determine whether a new diagnosis of pediatric diabetes can be successfully managed in a day hospital model.

**Design, Setting, and Participants:**

This quality improvement study used retrospectively collected data on pediatric patients with a new diagnosis of diabetes who completed an inpatient program for education and insulin titration prospectively compared with patients completing a diabetes day hospital program. Baseline data were collected over 12 months (January-December 2015) and intervention data collected over 14 months (March 2016-May 2017). The study was conducted at a single institution and judged as a nonhuman participant project. The referral local base included a 100-mile radius. Patient inclusion was a new diagnosis of diabetes, age 5 years or older, and no biochemical evidence of diabetic ketoacidosis. Ninety-six patients completed the day hospital program and 192 patients completed an inpatient program.

**Exposures:**

All patients received 2 consecutive days of insulin titration and education in either a day hospital or inpatient setting.

**Main Outcomes and Measures:**

Primary outcomes included the mean length of stay, patient charge, and insurance denial/reimbursement rates. The hypothesis was that a day hospital program would be associated with a reduced length of stay, which would directly affect patient charges and insurance denials.

**Results:**

Among the 96 day hospital patients, the mean (SD) age was 12.2 (4.7) years (range 5-20.3), with no patients experiencing diabetic ketoacidosis or hypernatremia. Among the 192 inpatient patients, the mean (SD) age was 9.4 (4.7) years (range, 1.6-20.1). The mean (SD) length of stay reduction in the day hospital was 46 (14.1) to 14 (5.1) hours. The mean day hospital patient charge was $2800, compared with a mean (SD) baseline carge of $24 103 ($9401). Within the first year, there was a cumulative reduction in patient charges of more than $2.1 million.

**Conclusions and Relevance:**

This study’s findings suggest that a diabetes day hospital setting was associated with reductions in length of stay and patient charges, with an increase in insurance reimbursements and a decrease in insurance denials. This study demonstrates an effective way to streamline new-onset diabetes education, which may reduce length of stay and patient charges. Reimbursement rates for patients with a new diagnosis of diabetes increased from 52% to 72% and reimbursement denial rates decreased from 80% to 0%.

## Key Points

**Question:**

Is a diabetes day hospital program for those with newly diagnosed diabetes associated with improved cost-effectiveness compared with a traditional inpatient program?

**Findings:**

In this quality improvement study, 96 patients with a new diabetes diagnosis were treated in a day hospital setting and 192 were treated in an inpatient setting. Cost-effectiveness was seen with patient charge reductions, a decreased length of stay, increased insurance reimbursements, and the elimination of insurance denials.

**Meaning:**

These findings indicate that treating the medical and educational needs of those with a new diagnosis of type 1 diabetes may be more cost-effective in a day hospital setting compared with hospital admissions.

## Teaser

This quality improvement study evaluates the success of treating children with type 1 diabetes in a day hospital vs a traditional inpatient model.

## Introduction

Discoveries such as the use of injectable insulin in the 1920s and the development of at-home monitoring tools (ie, glucometer) in the 1970s revolutionized care for patients with type 1 diabetes. Over time, these improvements and others have made physician involvement with diabetes management an increasingly outpatient endeavor.^1^ In recent years, the initial inpatient treatment for patients with new-onset type 1 diabetes do not have diabetic ketoacidosis (DKA), defined as a bicarbonate level of at least 18 mEQ/L (to convert to millimoles per liter, multiply by 1) or negative urine ketone results, has not been shown to provide long-term benefits despite being more costly than outpatient care. This has led to a higher frequency of insurance coverage denials for patients with a new diagnosis of diabetes. A study looking at the long-term outcomes of patients age 1 to 18 years with new-onset type 1 diabetes who received insulin initiation education in an outpatient vs inpatient setting showed no statistically significant difference in hemoglobin A_1c_ values 1 year postdiagnosis.^2^ Furthermore, as expected, charges were lower for the same health care services (ie, diagnostic testing, hospital care, supplies, and pharmacy) for those treated in an outpatient setting.

Our institution (University of Cincinnati College of Medicine; Cincinnati, Ohio) historically required all patients with a new diagnosis of type 1 diabetes to be admitted for insulin management and education. During a typical new-onset admission for patients in or not with DKA, insulin doses would be titrated and comprehensive education would be performed over 2 to 3 days (8 am to 5 pm). This process often resulted in lengthy admissions for patients who did not present with DKA and who were otherwise medically ready for discharge on admission. Thus, as patient charges rose, insurance reimbursement denials increased. A comparison looking at our hospital, which used inpatient treatment for new onset diabetes, and peer institutions, which used outpatient treatment for new onset diabetes, was completed using the 2015 Pediatric Health Information System. These data are a comprehensive database containing clinical and financial data on patients who had a diagnosis of diabetes from more than 456 freestanding pediatric hospitals across the United States. Comparison data revealed that our institution’s average patient charge for a primary diagnosis of type 1 diabetes was $4000 more per visit than patients at peer institutions (median of $19 900 [interquartile range, $14 740-$15 900] vs median of $15 900 [interquartile range, $10 360-$24 170]; *P* < .001), with length of stay accounting for 40% of the total patient charge. We then verified data through an internal institutional review of patients treated for a new diagnosis of diabetes from January 2015 to December 2015. Internal data revealed a length of stay totaling 60 hours and readmission rates, although not significantly different, trended lower than peer institutions (30-day readmission rate: 3% vs 15%; *P *=* *.06). From these data, we concluded that the large discrepancy seen in patient charges needed to be improved while maintaining lower readmission rates. The study was started by designing and conducting a 2-phase project, focusing on reducing length of stay. Phase 1 and its lead into phase 2 is described next.

Phase 1 included 113 patients with a new diagnosis of type 1 diabetes who did not have DKA. Interventions focused primarily on eliminating inefficiencies in our institution’s original inpatient process to lower the total length of stay. Patients were taught only survival skills associated with the management of type 1 diabetes (ie, blood glucose level monitoring, carbohydrate counting and dosing, long-acting insulin administration, and hypoglycemia and ketone corrections). Reductions in average patient charges by $1700 and in average length of stay by 12 hours were seen.

The phase 1 results were most beneficial for the subgroup of patients with a new diagnosis of type 1 diabetes who had DKA because it reduced their overall admission charges and they all required at least 1 overnight admission. However, this new process continued to require even the patients without DKA to remain in the hospital overnight while awaiting the conclusion of education the following day (average 10-15 hours of waiting). In addition to this underproductive time, it was also recognized that time spent in the hospital could expose patients to unnecessary risks, including hospital-acquired infections. Thus, to minimize unnecessary time spent in the hospital, it was determined that patients not in DKA could be best served by returning home between the 2 consecutive days of structured education. This led to the development of the second phase of the project, forming a diabetes day hospital for patients with a new diagnosis of type 1 diabetes who did not have DKA, which is described later.

## Methods

### Setting

Our institution is a large, urban pediatric academic medical center. On a yearly average care is provided for more than 33 000 inpatient admissions, 179 000 urgent care and emergency department visits, and 984 000 specialty outpatient visits. Approximately 35 000 surgical procedures are performed at the main campus and 10 neighborhood locations in surrounding communities. Our institution’s reach includes a local referral base of a 100-mile radius in addition to patients from all 50 states and 107 countries. Our institution cares for more than 2000 patients with type 1 diabetes and approximately 150 to 200 patients with new-onset type 1 diabetes annually. The Cincinnati Children’s Hospital Medical Center institutional review board judged this project to be not human participants research, and therefore consent was not recommended.

### Day Hospital Formation

Interventions in phase 2 focused on fundamentally altering the process for new-onset type 1 diabetes education by implementing a 2-day day hospital model in which patients received education during the day and used the skills learned during those sessions to care for themselves at home overnight. The program was designed with the expectation that nearly all patients would be administered basal-bolus injection therapy.

Patients with new-onset diabetes typically present either to their primary care clinician, an outside emergency department, or our institution’s emergency department. The clinicians in these locations notify the inpatient endocrinology attending physician or fellow physician of the patient’s clinical information. A safety protocol to categorize all patients with new-onset diabetes was developed during the initial planning step for use by the inpatient endocrinology team (Figure). Patients who were at least age 5 years (because of the higher risk of adverse outcomes after taking insulin) and who did not have a social situation making the day hospital unsafe (ie, primary guardian not present) or impractical (ie, transportation concerns, interpreter services needed) were categorized as appropriate for day hospital treatment. After a new patient was determined safe for treatment at the day hospital by the division of endocrinology attending physician or fellow, the inpatient team physicians (attending, fellow, and residents) collaborated to determine insulin doses for day hospital day 1. By default, any patient not categorized as inappropriate for treatment in the day hospital was automatically admitted and treated according to the phase 1 process described previously.

**Figure.**
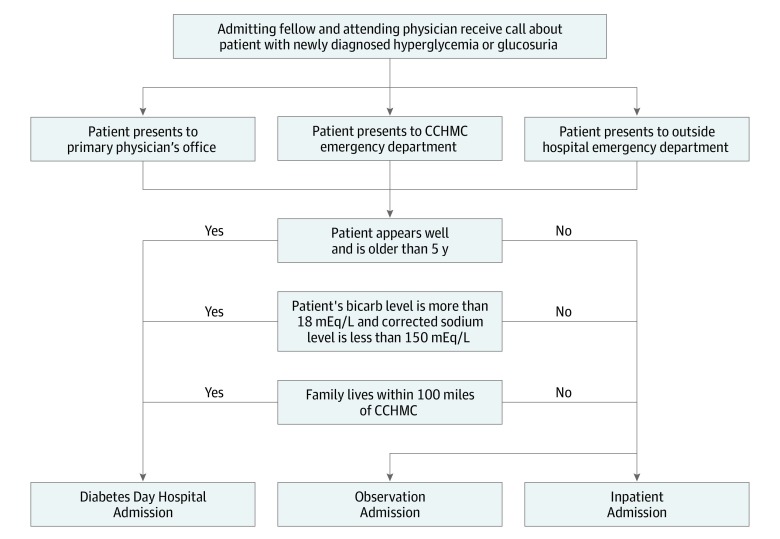
New-Onset Type 1 Diabetes Admission Decision Simplified Care Algorithm Bicarb indicates bicarbonate; CCHMC, Cincinnati Children’s Hospital Medical Center. To convert bicarb and sodium to millimoles per liter, multiply by 1.

To help prepare for the day hospital, the team created a handout that was accessible online to all emergency departments and primary care clinicians. This document was printed and presented to patients and families before leaving their primary care office or the emergency department (eFigure 1 in the Supplement). The handout includes an introduction to diabetes and practical information about the day hospital program.

#### Day 1

eFigure 2 in the Supplement shows the schedule for day 1. The primary education goals were for the family to demonstrate proficiency in the following topics: diabetes overview, glucometer testing, carbohydrate counting, insulin injections, ketone testing, and consuming carbohydrates to treat hypoglycemia. A social work assessment of coping, needs, and barriers was also an essential component of day 1. With each meal, the medical team evaluated blood glucose values and adjusted bolus insulin dosing as necessary. At the end of day 1, the first dose of basal insulin (usually glargine) was administered before discharge. Families were instructed to check glucose values before meals, overnight, and on waking in the morning. They were provided bolus insulin for carbohydrates eaten at home but were not yet expected to administer insulin to correct high blood glucose levels. They were provided contact information for the physician on call and instructed to call overnight with recurrent hypoglycemia or ketonuria.

#### Day 2

The schedule for day hospital day 2 is outlined in eFigure 3 in the Supplement. The family needed to demonstrate proficiency in calculating insulin doses for hyperglycemia and glucagon administration. Families calculated insulin doses at all mealtimes during day 2 and had to demonstrate that they were able to do this autonomously before they were discharged home. The medical team monitored glucose values and adjusted insulin doses as needed before discharge.

#### Follow-up

At the completion of day 2, patients/families were expected to possess basic knowledge and skills to safely manage diabetes at home. However, most families need to review concepts and build on them to optimally manage their diabetes long-term. Therefore, we asked all families to call and review glucose trends with staff daily following discharge until skill competency, resolution of erratic glucose values (ie, large swings between hyperglycemia and hypoglycemia), and minimal ketonuria were appreciated. Additionally, families were scheduled to have a follow-up education session at their first diabetes clinician visit 2 to 3 weeks after discharge.

### Outcomes and Data Collection

An internal data review was performed on baseline and postintervention data. Unlike the Pediatric Health Information System data described earlier for our preliminary comparison, internal baseline data eliminated all patients admitted with type 1 diabetes as a secondary diagnosis to only capture patients with a new diagnosis of diabetes. Additionally, admission data were used instead of billing data to improve precision. The primary outcome measures used for internal baseline data included patient charges and length of stay. Thirty-day readmission rates, after-hours calls to the division of endocrinology, and emergency department visits at our institution postdischarge were tracked and used as balancing measures.

Data from 96 patients who completed the standard 2-day program were compared with 192 patients from the baseline period who would have qualified for the day hospital program had it been in place at the time of their diagnosis (Table). Day hospital charges were standardized for all patients, as our institution receives the same reimbursement for every patient who participates in the day hospital program. Data were collected on all after-hour calls to the division of endocrinology occurring between 4:30 pm and 8:30 am each day, except overnight Saturdays through Sunday morning. Overnight Saturday calls were excluded because of limitations in automatic data collection. Baseline after-hours call data collection began during phase 1 and included the 35 weeks before starting phase 2. Postintervention data collection included the first 52 weeks after the implementation of the day hospital program (phase 2).

**Table.** Demographic Characteristics of Baseline and Postintervention Populations

Characteristic	No. (%)		Total, No.	Mean Age (Range), y
	Male	Female		
Baseline	114 (59)	78 (41)	192	11.5 (5-21.6)
Postintervention	53 (55)	43 (45)	96	12.2 (1.4-20.3)

### Data Analysis

The length of stay for 96 patients (39 female [41%]) who completed the standard 2-day program were compared with 192 patients (86 female [45%]) from the baseline period using a 2–independent sample *t* test. A 1-sample *t* test was used to compare the baseline cost with the fixed postintervention cost. The emergency department visit rate within 30 days of the day hospital program was compared with the baseline period using the Fisher exact test. Analysis was performed using SAS, version 9.4 (SAS Institute). The after-hours call data was compared using a 2–independent sample *t* test. A 2-tailed α level of .05 was used for all statistical tests.

## Results

This is a prospective project comparing retrospectively collected inpatient data with newly acquired day hospital data. Patients admitted to the diabetes day hospital were older than 5 years (mean [SD] age, 12.2 [4.7] years [range 5-20.3]), did not have DKA, and did not have significant hypernatremia. The metabolic profile required for admission to the day hospital included a ketonuria level of less than 60 mg/dL and/or bicarbonate level greater than 18 mEq/L and serum sodium level not greater than 155 mEq/L (to convert sodium to millimoles per liter, multiply by 1). Patients admitted to the inpatient unit were younger than 5 years or were older than 5 years and did not meet the metabolic criteria for admission to the day hospital (mean [SD] age, 9.4 [4.7] years [range, 1.6-20.1]). Following implementation of the day hospital program, the mean (SD) length of stay was reduced significantly from 46 (14.1) to 14 (5.1) hours for patients did not have DKA and who qualified for the 2-day day hospital program (*t*_286_ = 21.31; *P* < .001). The mean charge for the 2-day day hospital was $2800 compared with a mean (SD) baseline of $24 103 ($9401). This produced a mean savings of more than $21 000 per admission. Specifically, in the first year, there was an average reduction in length of stay of 30 to 35 hours and a cumulative reduction in patient charges of more than $2.1 million. Reimbursement rates for patients with a new diagnosis of diabetes increased from 52% to 72% and reimbursement denial rates decreased from 80% to 0%. Readmission rates for patients with new-onset diabetes remained essentially the same (30-day: 0.3% to 0.5%; *P* = .83). Emergency department visits within 30 days of completing the day hospital program were comparable with the baseline 30-day emergency visit rate (0.2% and 0.9%, respectively; *P* = .18). There was no statistically significant difference in the mean (SD) number of after-hours calls to the division of endocrinology between phase 1 (29.5 [7.2] calls/week) and phase 2 (29.1 [7.1] calls/week; *P* = .80).

## Discussion

Data from a Cochrane review^3^ suggested that adequate outpatient/home management of type 1 diabetes in children at diagnosis does not lead to any disadvantages in terms of metabolic control, acute diabetic complications and hospitalizations, psychosocial variables and behavior, or total costs.^3^ Jasinski et al^2^ reported on metabolic outcomes, health care use, and costs among children with new-onset type 1 diabetes who received their initial education and care in an outpatient pediatric endocrinology clinic or a hospital. There were no statistically significant differences in metabolic outcomes between groups at 1 year^2^ and 2 years^4^ postdiagnosis. However, the mean total patient charges per child were 2.4 times higher in the inpatient group ($12 332 vs $5053; *P* < .001) at 1 year postdiagnosis.^2^ Semistructured interviews with parents and children participating in the Delivering Early Care In Diabetes Evaluation study showed that home treatment was initially more preferable than hospital management for most parents but both settings were acceptable.^5^ Our study demonstrated a model for treating newly diagnosed type 1 diabetes in which real-life simulation is possible, newly learned skills can be practiced at home with reinforcement the next day, and a standardized cost for all treatment (diabetes stabilization and education alone vs the addition of more modest therapies [ie, short courses of intravenous fluids]) can be declared up front.

This study’s initial attempt to reduce patient charges and the length of stay associated with management of new-onset type 1 diabetes focused primarily on eliminating inefficiencies in the inpatient process. After making inpatient changes to streamline education and improve efficiency, only small reductions in patient charges and length of stay were achieved; however, the insurance denial rate remained high. Following implementation of a day hospital program that allowed patients without DKA or those recovered from DKA to go home at night, we achieved large reductions in the length of stay and patient charges, with an associated increase in insurance reimbursements (20% absolute increase) and decrease in insurance denials (100% reduction). Over a 14-month period (March 2016-May 2017), readmission rates remained low and comparable with emergency department visits, and after-hours calls to the division of endocrinology remained low. This indicated an adequate parental and patient understanding of basic diabetes knowledge, allowing families to adequately perform diabetes care at home.

As expected, length of stay was the primary driver of decreasing patient charges. Eliminating the patient’s overnight stay, when minimal education occurred and few management decisions were made, was the primary driver for decreasing the length of stay. Sending patients home for the night did not slow their rate of learning or decrease their satisfaction. In fact, our anecdotal experience suggested that they felt more empowered by going home. This became evident when families were seen completing day 2 education plus a full review of diabetes care an average of 6 to 8 hours faster than they completed day 1 education. Additionally, the emergency department was not overwhelmed with recently discharged patients who were confused about their care (inpatient readmission rates comparable with the emergency department visit rate).

Since opening the diabetes day hospital, we have been able to expand services to other populations. We have developed a modified 1-day program for patients with mild new-onset type 2 diabetes who need primarily education and less intensive medical treatment. We also now have increased capacity on the inpatient diabetes floor for more urgent or complicated diabetes admissions (ie, DKA, hypoglycemia, total pancreatectomy, and patients receiving insulin pump therapy after surgery).

### Limitations

Our program demonstrated that care for children with a new diagnosis of type 1 diabetes who do not have DKA can have successful outpatient care, with patient charges standardized. Our diabetes day hospital charges remain consistent for all patients who do not have DKA regardless of the additional care they may require (ie, education only or intravenous fluids and education). The day hospital model could be adopted by many other organizations; however, we understand that this generalizability may be limited by differences in insurance reimbursement policies throughout the country and each hospital’s community reach. We are fortunate to serve a large local community (a more than 100-mile radius) but also treat a referral base encompassing all 50 states and more than 100 countries. Additionally, we acknowledge that our success was enhanced by the availability of unused outpatient bed space during daytime hours, ample access to a large education team (certified diabetes educators and registered dieticians), bedside nursing staff committing extra time to program development, and an internal liaison team who provided outreach and education to local primary care and emergency department clinicians.

## Conclusions

Following a coordinated and multidisciplinary implementation of a day hospital program for patients with new-onset type 1 diabetes who were not in DKA, we achieved a significant reduction in length of stay and patient charges. This allowed our institution to eliminate insurance denials and improve reimbursement. Moreover, these outcomes were achieved without an increase in readmissions or after-hours call volume. Advantages of the program also included adaptability to a model for type 2 diabetes and increased inpatient bed space for more urgent or complex diabetes admissions. Therefore, the day hospital program implemented at our institution may be used as a sustainable model for achieving cost-effective, safe, and patient-centered care for youth with new-onset type 1 diabetes.

## Notes

Supplement. **eFigure 1.** Parent/Family Welcome Letter **eFigure 2**. Education Schedule Day 1 **eFigure 3. **Education Schedule Day 2
