# Supplementary material for: Assessment of a Day Hospital Management Program for Children With Type 1 Diabetes
Source: JAMA Netw Open. 2020 Mar 3;3(3):e200347. doi: 10.1001/jamanetworkopen.2020.0347 (PMC7054842; doi:10.1001/jamanetworkopen.2020.0347)
Supplement: Supplement. — eFigure 1. Parent/Family Welcome Letter eFigure 2. Education Schedule Day 1 eFigure 3. Education Schedule Day 2 [file jamanetwopen-3-e200347-s001.pdf]

Supplementary Online Content

Lawson S, Redel JM, Smego A, et al. Assessment of a day hospital management program for children with type 1 diabetes. *JAMA Netw Open*. 2020;3(3):e200347. doi:10.1001/jamanetworkopen.2020.0347

eFigure 1. Parent/Family Welcome Letter
eFigure 2. Education Schedule Day 1
eFigure 3. Education Schedule Day 2

This supplementary material has been provided by the authors to give readers additional information about their work.

© 2020 Lawson S et al. *JAMA Network Open.*

eFigure 1: Parent/Family Welcome Letter

Welcome to the
Diabetes Center Day Hospital

(two consecutive day visits)

Our institution’s Diabetes Center is dedicated to providing the highest level of comprehensive care for your child. The purpose of attending our Day Hospital program is to teach you how to safely care for yourself/your child’s diabetes. During the Day Hospital visits, we will help you understand diabetes, how to use insulin, and how to incorporate the care for you/your child at home. Our providers and education team look forward to helping you on this journey!

What is Diabetes?

Diabetes is a chronic illness. With proper care and treatment, your child will live a healthy and active life. Diabetes occurs when the body does not have enough insulin or is not able to use insulin properly. In most cases, treatment **must** include daily insulin injections. There is nothing that you could have done to prevent your child from being diagnosed with diabetes.

⌋ ***Please read the following suggestions before coming to the day hospital visit:***

- **The legal guardian must always come with their child to the Day Hospital. We will be reviewing important information on your child’s care.**
- **Make sure the patient drinks plenty of water or sugar-free, caffeine-free drinks.**
- **If vomiting or stomach pain occurs, or you feel your child is getting worse, call your primary care doctor or go to the closest Emergency Department.**

⌋ ***Planning for your Day Hospital Appointment:***

- **We will provide meals for your child. Your child will receive breakfast, lunch, and an afternoon snack. Please do not feed your child breakfast prior to arrival so that blood work can be completed, if needed.**
- **Legal guardians are expected to bring their own food due to limited time. A microwave and refrigerator are available for your use**
- **Bring your insurance and prescription cards each day.**
- **Only the legal guardian(s) receiving education and the patient are permitted to be present for appointments. However, for younger patients, another adult or older child may come to stay with the patient while you are getting education in a separate room.**
- Think about bringing some toys or games to keep your child occupied while you receive education. There is a TV and a limited movie selection in their room.

© 2020 Lawson S et al. *JAMA Network Open.*

- Please limit cell phone use during your education sessions so we can cover all the information during the time you are there.
- Please plan for your childcare and employment needs. We can provide you with school and work excuses if needed.

Location and Parking

- The Day Hospital is at the main campus of our institution.
- Park in the visitor parking garage.

Registration

You must register in Admitting each day you come. If you have questions please call Admitting.

What to Expect at Day Hospital Visits One and Two:

Daily Schedule

7:30 am	Register in Admitting and come up to the Day Hospital.
8:00-10:00 am	Complete check in process with medical team, meet the doctors, eat breakfast, participate in medical rounds.
10:00 am-4:00 pm	Attend scheduled appointments each day with the diabetes education team members (nurse educator, dietitian, and social worker).

** Your training will start as soon as you arrive and will continue through mealtimes.

** Remember we will provide meals and snacks for your child. You will need to bring food for the caregivers attending education.

- Education will last until about 4:00 pm. The ending time may vary depending on each family's educational needs. If your child is more ill than expected, they could be admitted overnight.
- During your stay, you will receive:
    - written educational materials that will help you to manage your child's diabetes
    - diabetes supplies that will last you until prescriptions are ready at your pharmacy

---

Helpful Resources:

Below is a link that will give you more information on diabetes until we are able to meet with you at Day Hospital.

© 2020 Lawson S et al. *JAMA Network Open.*

eFigure 2: Education Schedule Day 1

**DAY ONE**	
7:30 AM – 10 AM	1. Check in downstairs/walk to room<br>2. Residents check in<br>3. RN check in (vitals, labs, etc)<br>4. Pt selects breakfast(from standard tray), lunch and snack for the day<br>5. Breakfast – RN shows parents how to test blood sugar (ketones if appropriate), draw up & give insulin (COMPLETED BY 9AM)<br>6. Rounds<br>7. Insurance verification<br>8. Potential time with Child Life
10 AM -12 PM	EDUCATION – RN EDUCATOR<br>1. What is diabetes – signs and symptoms<br>2. Blood glucose testing – meter set up (assistance from 2nd educator if class)<br>3. Insulin injection (how to administer)<br>4. Details of Insulins (storage, duration/peak, etc).<br>5. Testing ketones<br>6. Treating low blood sugar
12:00 – 1:00 PM	LEARNING LUNCH<br>1. Parents check blood sugar with nurse supervision/guidance<br>2. Parents give injection with nurse supervision/guidance<br>3. Eat Lunch (parents & patient)
1:00-2:30 PM	EDUCATION – RD EDUCATOR<br>1. Carbohydrate Counting<br>2. Calculations for carbohydrate bolus only<br>3. Questions with educator for Day one
2:30 PM- 3:15 PM	SOCIAL WORK<br>1. Explore, process, and normalize feelings about diagnosis<br>2. Gather information on school, school nurse, daycare, etc.<br>3. Discuss insurance and any potential concerns. Provide information on BCMH.
3:15 PM – 4 PM	1. Team confers to determine if patient is safe to go home or needs to be admitted overnight<br>2. Obtain breakfast menu for Day 2<br>3. Parents give Lantus injection<br>4. Afternoon snack –<br>5. Parents check blood sugar with nurse supervision/guidance<br>6. Parents give injection with nurse supervision/guidance<br>7. Discharge – expectation letter/info for Day 2 of education<br>8. Potential time with Child Life

© 2020 Lawson S et al. *JAMA Network Open.*

eFigure 3: Education Schedule Day 2

**DAY TWO**	
7:30 AM – 9:30 AM	1. Check in downstairs/walk to room<br>2. Residents check in<br>3. RN check in (vitals, labs, etc)<br>4. Pt selects breakfast*, lunch and snack for the day<br>5. Breakfast – parents test/give insulin<br>6. Rounds<br>7. Potential time with Child Life
9:30 AM - 11/11:15 AM	EDUCATION – RD EDUCATOR<br>1. Insulin calculation in entirety<br>2. No Correction Rules<br>3. Night time vs Daytime correction factor
11/11:15 AM – 12:00 PM	SOCIAL WORK<br>1. Process feelings about transitioning back to home and school<br>2. Review and provide school packet and community resources
12:00 PM - 1:00 PM	LEARNING LUNCH<br>1. Parents check blood sugar with nurse supervision/guidance<br>2. Parents give injection with nurse supervision/guidance<br>3. Eat Lunch (parents & patient)
1-2 PM	EDUCATION – RN EDUCATOR<br>1. Glucagon<br>2. Discuss calling in for insulin dose adjustments<br>3. Expectation letter/info for Day 3 & 4 of education
2 PM- 3 PM	1. One on one question time with one of two educators teaching Day 2<br>2. Evaluate comprehension of calculations/No correction rules/all topics<br><br>2-2:30 PM –RN educator<br>only 2:30-3 PM – RD<br>educator joins
3PM – 4 PM	1. Team confers to determine if patient is safe to go home or needs to be admitted overnight<br>2. Afternoon snack –<br>Parents check blood sugar with nurse supervision/guidance<br>parents give injection with nurse supervision/guidance<br>3. Discharge – AVS, instructions for f/u<br>4. Potential time with Child Life

© 2020 Lawson S et al. *JAMA Network Open.*

© 2020 Lawson S et al. *JAMA Network Open.*
